# Ein ungewöhnlicher Fall: Fraktur des Hammergriffs durch Fingermanipulation

**DOI:** 10.1007/s00106-024-01503-1

**Published:** 2024-07-22

**Authors:** Peter M. Mair, Thomas Mayr, Georg Sprinzl, Astrid Magele

**Affiliations:** 1 Klinische Abteilung für Hals‑, Nasen‑, Ohrenkrankheiten, Universitätsklinikum St. Pölten, Dunant-Platz 1, 3100 St. Pölten, Österreich; 2 Karl-Landsteiner-Privatuniversität für Gesundheitswissenschaften, Krems, Österreich Dr. Karl-Dorrek-Straße 30, 3500

**Keywords:** Gehörknöchelchenkette, Glasionomerzement, Schallleitungsschwerhörigkeit, Otologie, Audiologie, Ossicular chain, Glass ionomer cement, Conductive hearing loss, Otology, Audiology

## Abstract

Die isolierte Hammergrifffraktur ist eine seltene, aber klinisch relevante Verletzung im Mittelohr, die zu einer einseitigen Hörminderung führt. Ursachen können akute Druckveränderungen oder traumatische Ereignisse sein. Verschiedene Therapieansätze wie Tympanoplastik, Transposition von autologem Material oder Applikation von Knochenzement werden diskutiert. In dieser Kasuistik wird eine 46-jährige Patientin mit einseitiger Hörminderung nach Manipulation im Ohr beschrieben. Klinisch zeigen sich eine Achsenfehlstellung des Hammergriffs und eine Schallleitungshörminderung in der Audiometrie. Die Verdachtsdiagnose lautet Unterbrechung der Gehörknöchelchenkette. Im Rahmen einer Tympanoskopie wird die Diagnose isolierte Hammergrifffraktur gestellt. Es wird in weiterer Folge Glasionomerzement zur Rekonstruktion des Hammergriffs verwendet. Postoperativ verbessert sich die Hörfunktion signifikant, mit einer vollständigen Remission der Hörminderung nach vier Monaten. Diese Kasuistik zeigt die Bedeutung einer umfassenden Diagnostik und Erfahrung des Operateurs bei der Auswahl der Therapie.

## Abstract (en)

An isolated fracture of the handle of the malleus is a rare entity in otorhinolaryngology and manifests clinically as acute-onset unilateral hearing loss. Several factors may cause this injury, including acute barotraumatic pressure changes or traumatic events. Various therapeutic approaches such as tympanoplasty, autologous graft, or application of bone cement are discussed. We report the case of a 46-year-old female patient who developed acute hearing loss in her left ear after finger manipulation. Clinical evaluation revealed axial displacement of the handle of the malleus and audiometry indicated conductive hearing loss. After otoscopy, audiometry, and computed tomography, tympanoscopy was indicated due to suspicion of ossicular chain disruption. Intraoperatively, an isolated fracture of the handle of malleus was found, which was treated with glass ionomer cement. Following postoperative examination, there was progressive improvement in the acoustic transmission component, such that a normal hearing threshold was observed 4 months postoperatively. This case report underlines the importance of precise diagnosis and individualized treatment for rare middle ear injuries.

Die isolierte Hammergrifffraktur stellt eine seltene, aber klinisch relevante Herausforderung in der HNO-Heilkunde dar. Verschiedene Faktoren können diese Verletzung des Mittelohrs auslösen. Dazu zählen akute barotraumatische Druckveränderungen oder traumatische Ereignisse. Als Folge präsentiert sich in Abhängigkeit vom Schweregrad der Unterbrechung der Gehörknöchelchenkette eine Beeinträchtigung der Schallleitungsfähigkeit, welche von Patienten als akute, einseitige Hörminderung wahrgenommen wird.

Aufgrund der geringen Fallzahl gibt es verschiedene Therapieansätze der isolierten Hammergrifffraktur:

- *Tympanoplastik Typ III* - Partial Ossicular Replacement Prosthesis (PORP) und Total Ossicular Replacement Prosthesis (TORP) [1–3]
- *Transposition von autologem Material* [4–6]
- *Applikation von Knochenzement* [7–9].

## Falldarstellung

Eine 46-jährige Patientin wurde in unserer HNO-Ambulanz vorstellig mit dem Leitsymptom einer einseitigen Hörminderung links. Nach Manipulation mit dem Finger im linken Ohr habe die Patientin einen stechenden Schmerz verspürt, gefolgt von einer akuten, streng linksseitigen Hörminderung.

## Anamnese und Befund

Im Rahmen der klinischen Untersuchung zeigt sich unter dem Mikroskop ein blander äußerer Gehörgang. Das Trommelfell präsentiert sich reizlos und intakt, jedoch fällt sofort eine Achsenfehlstellung des Hammergriffs auf. Die zentrale Trommelfelleinziehung durch den Umbo fehlte, da sich der Umbo in Richtung des äußeren Gehörgangs vorwölbt. Der Stimmgabeltest ergibt: Weber lateralisiert nach links (betroffenes Ohr), Rinne links negativ, rechts positiv.

**Abb. 1:**
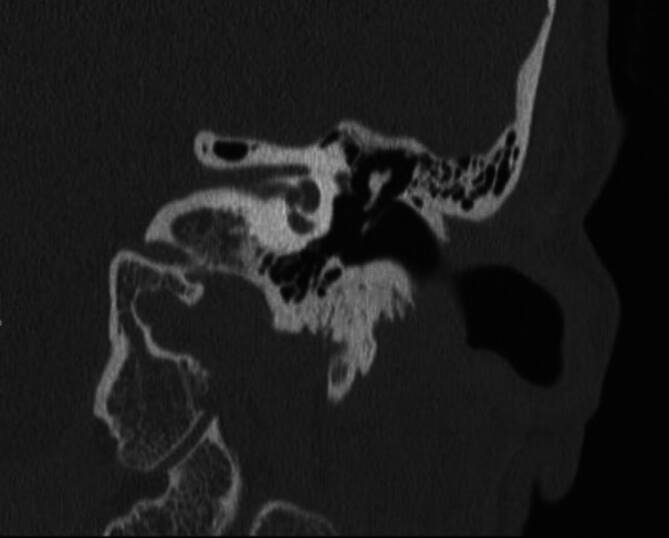
Koronare Darstellung der Gehörknöchelchenkette in der Felsenbein-CT

Eine Computertomographie (CT) des Felsenbeins wird durchgeführt, in welcher sich im Seitenvergleich der Hammergriff links deutlich kürzer zeigt und sich nicht vollständig bis zum Ansatz am Trommelfell abgrenzt. Am Ansatz des Hammergriffs im Trommelfell ist eine kleine knöcherne Struktur abgrenzbar. Sonst zeigt sich die Gehörknöchelchenkette regulär und intakt (Abb. 1).

**Abb. 2:**
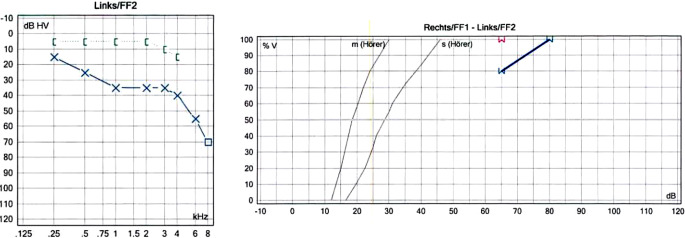
Ton- und Sprachaudiogramm präoperativ

In der tonaudiometrischen Untersuchung zeigt sich rechtsseitig ein unauffälliger Befund, während auf der linken Seite bei regulärer Knochenleitungskurve ein Air-Bone-Gap von bis zu 35 dB besteht, insbesondere in den mittleren und höheren Frequenzen. Im Sprachaudiogramm manifestiert sich die asymmetrische Hörminderung als reduziertes Sprachverständnis von 20 % bei 65 dB (Abb. 2).

## Verdachtsdiagnose

Leicht- bis mittelgradige Schallleitungshörminderung links mit V. a. Unterbrechung der Gehörknöchelchenkette.

## Therapie und Verlauf

Aufgrund der mittel- bis hochgradigen Schallleitungsschwerhörigkeit und der computertomographisch suszipierten Kettenunterbrechung wird die Indikation zur Tympanoskopie mit gegebenenfalls Rekonstruktion der Gehörknöchelchenkette gestellt.

**Abb. 3:**
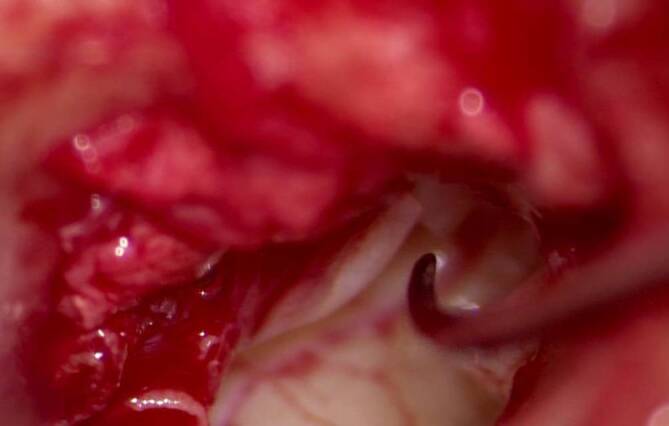
Spitze der Nadel zeigt auf die Frakturlinie

In Allgemeinanästhesie wird nach Eingehen über den Heermann’schen Gehörgangsschnitt der tympanomeatale Lappen ausgelöst. Nach Eingehen unter die Trommelfellebene wird das Mittelohr inspiziert, wo der luxierte, am Trommelfell fixierte Hammergriff erkannt wird. Mittels Bewegen des Hammerkopfes kann eine Fortleitung der Schwingung bis hin zum Stapes beobachtet werden. Somit kann die Diagnose „isolierte Hammergrifffraktur“ gestellt werden (Abb. 3 und 4).

**Abb. 4:**
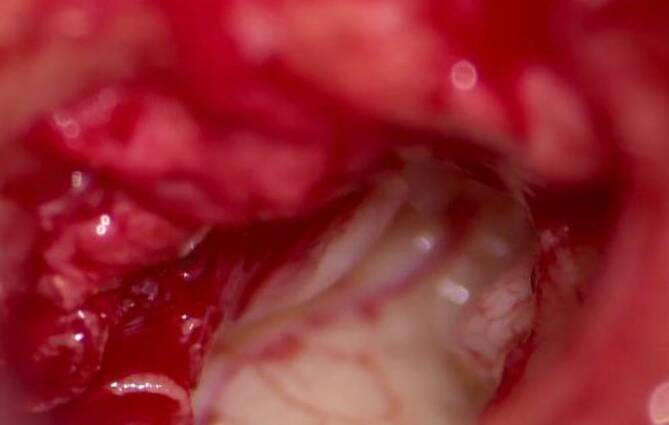
Frakturlinie ohne Nadel

**Abb. 5:**
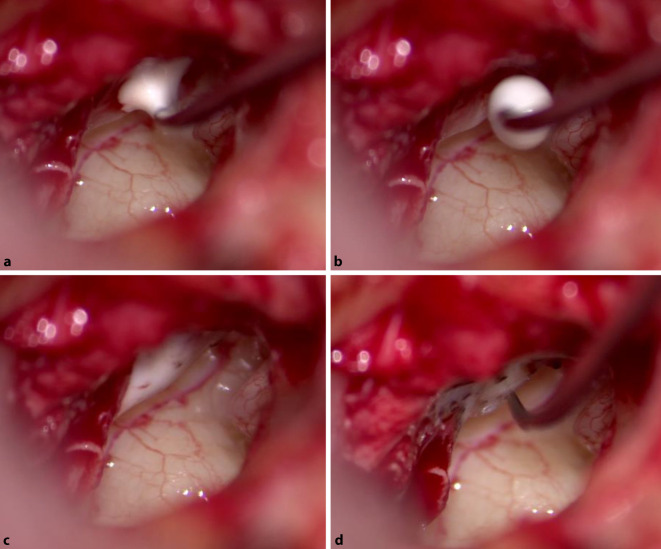
Applikation von GIC

In weiterer Folge wird intraoperativ die Entscheidung getroffen Glasionomerzement (GIC) mit einer Nadel auf die Fraktur zu applizieren (Abb. 5a) und präzise entlang des Hammergriffs bis hin zum Hammerkopf zu verteilen (Abb. 5b, c), sodass eine vollständige Rekonstruktion des Hammergriffs erzielt werden konnte (Abb. 5d).

Ursprünglich wurde GIC für die Zahnheilkunde in den 1970er-Jahren entwickelt. Im Jahr 1989 wurde die Anwendung von GIC in der HNO-Heilkunde erstmals dokumentiert [10]. Vor allem in der rekonstruktiven Mittelohrchirurgie hat sich der Zement aufgrund einer hohen Biokompatibilität und einer einfachen Handhabung und Verarbeitung als biostabiles Knochenersatzmaterial etabliert. Das pulverförmige Silikatglas wird dabei mit Polyacrylsäure gemischt. Dadurch entsteht eine pastöse Substanz, die einfach auf einen Bruchspalt aufgetragen werden kann [11, 12].

Nach ungefähr 6 min Trockenzeit ist der Zement gehärtet, sodass die Bewegung am Hammergriff bis hin zum Stapes fortgeleitet werden kann. Damit ist die Kettenkontinuität wiederhergestellt.

Abschließend wird der tympanomeatale Lappen zurückgeschlagen, Ohrfolien werden eingelegt und der Gehörgang mit Gelita-Tamponaden aufgefüllt.

**Abb. 6:**
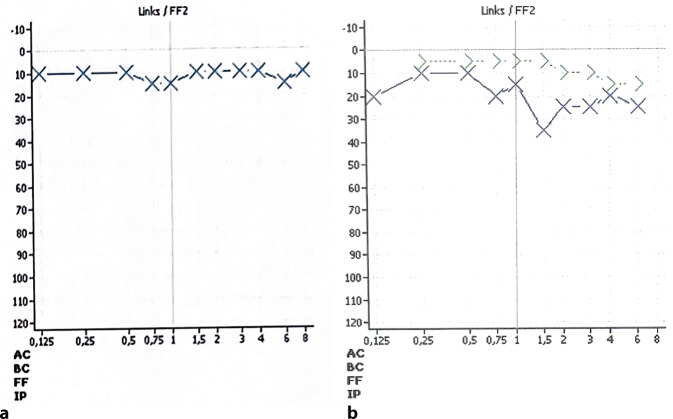
Audiometrische Kontrollen 2 Wochen (**a**) und 4 Monate (**b**) postoperativ

Am ersten postoperativen Tag zeigt die Knochenleitungsprüfung einen unveränderten Befund. Zehn Tage postoperativ wird die Detamponade durchgeführt. Es zeigt sich der tympanomeatale Lappen gut eingeheilt. Nach zwei Wochen wird erneut eine audiologische Kontrolle durchgeführt. Bei unveränderter Innenohrfunktion zeigt sich bereits eine deutliche Besserung des Air-Bone-Gaps in beinahe allen Frequenzen. Lediglich bei 1,5 kHz kann keine Besserung erzielt werden. Nach vier Monaten zeigt sich bereits eine Normakusis bei einer vollständigen Remission der Schallleitungshörminderung (Abb. 6).

## Diskussion

Die isolierte Hammergrifffraktur zählt zu den seltenen Defekten im Bereich des Mittelohrs, weshalb in der Literatur nur wenige Fälle beschrieben sind. Die häufigste Ursache ist die digitale Manipulation, andere Ursachen stellen Verletzungen durch Fremdkörper und (Baro‑)Traumata dar [1, 13]. In sehr seltenen Fällen führt unterdrücktes Niesen zu einer Hammergrifffraktur [8].

Die Verwendung von Glasionomerzement zur Behandlung der isolierten Hammergrifffraktur stellt eine erfolgreiche Methode dar. Im Vergleich zu anderen chirurgischen Eingriffen bietet diese interventionelle Technik mehrere potenzielle Vorteile, darunter eine einfache Anwendung, geringe Invasivität und eine schnelle Rehabilitation. Sowohl bei voroperierten Ohren als auch bei ungünstigen Schleimhautverhältnissen kann der GIC aufgrund der hohen Biokompatibilität verwendet werden [12]. Lübben et al. haben jedoch in einer 2001 publizierten Studie zytotoxische Wirkungen beobachtet, wenn GIC nicht sorgfältig vor Flüssigkeitskontakt geschützt wurde. Wenn der Zement vor Aushärtung mit Flüssigkeit in Kontakt kommt, kann es zur Lösung von Fluorid- und Aluminiumionen kommen [14]. Bei Kontakt mit Liquor cerebrospinalis (CSF) wurden 1994 zwei Fälle einer fatalen Aluminium-Enzephalopathie beschrieben [15, 16].

Aufgrund der Seltenheit dieser Krankheitsentität kann zum momentanen Zeitpunkt keine wissenschaftlich fundierte Aussage über die Überlegenheit eines Therapieansatzes der isolierten Hammerkopffraktur getroffen werden. In dem von uns beschriebenen Fall zeigt sich eine vollständige Rehabilitation der Schallleitungsschwerhörigkeit. Vier Monate postoperativ kann bei einer regulären Schallübertragung eine Normakusis gemessen werden. Dieses Ergebnis deckt sich auch weitestgehend mit einer Publikation von Zhao et al. [8]. In einer weiteren Fallstudie wurde die Fraktur mittels Knorpelbrücke geschient (Tragus-Knorpeltransposition). Dadurch besserte sich die Schallübertragung jedoch nur in den niederen Frequenzen, bei beinahe gleichbleibender Schallleitungsschwerhörigkeit ab 1 kHz [17]. Im Gegensatz dazu zeigt sich das postoperative Ergebnis nach einer Titan-PORP-Implantation: Hierbei wird eine Besserung der Schallübertragung in den niederen Frequenzen dokumentiert, ab 3 kHz kommt es jedoch zu einer Zunahme der Schallleitungsschwerhörigkeit [3].

Langzeitstudien sind jedoch unerlässlich, um die Langzeitergebnisse und Langzeitkomplikationen der Verwendung von GIC bei der Behandlung von isolierten Frakturen des Hammergriffs besser zu verstehen. Diese Studien könnten Aufschluss über potenzielle Probleme wie Materialdegradation, Lockerung des Zements oder andere Langzeitkomplikationen geben.

## Fazit für die Praxis

- Die isolierte Hammergrifffraktur stellt eine seltene Entität der Schallleitungshörminderung dar.
- Die Diagnostik umfasst die klinische Ohrmikroskopie, die computertomographische Darstellung der Gehörknöchelchenkette sowie eine audiologische Abklärung.
- In der vorliegenden Fallstudie wird eine erfolgreiche operative Versorgung der isolierten Hammergrifffraktur mittels Glasionomerzement dokumentiert.
- Die Anwendung dieser Methode bessert nicht nur die auditive Funktion durch Wiederherstellung der Schallleitungsfunktion des Mittelohrs, es lassen sich zudem aufwendigere chirurgische Eingriffe wie zum Beispiel die Tympanoplastik Typ III (TORP/PORP), die Implantation eines aktiven Mittelohrimplantats („active middle ear implant“, AMEI) oder eines Knochenleitungsimplantats (d.h. Bonebridge, Fa. MED-EL, Innsbruck, Österreich, oder Osia, Fa. Cochlear, Hannover, Deutschland) vermeiden.
- Aufgrund der geringen Fallzahlen und damit fehlenden klinischen Studien kann keine sichere Aussage über die Überlegenheit einer Therapieform getroffen werden.
- Entscheidend sind somit eine umfassende Diagnostik zum Ausschluss weiterer Pathologien im Mittelohr sowie die Erfahrung des Operateurs, um intraoperativ die bestmögliche Therapie zu wählen.
